# The lost balloon at midnight: a case report reveals the inevitability of heart team existence

**DOI:** 10.1186/s13019-023-02202-8

**Published:** 2023-04-06

**Authors:** Mohamed Elbayomi, Michael Weyand, Ehab Nooh, Frank Harig

**Affiliations:** Department of Cardiovascular Surgery, Friedrich-Alexander-University, Krankenhausstr. 12, 91054 Erlangen, Germany

**Keywords:** Percutaneous coronary intervention, Unstable angina pectoris, Cardiopulmonary bypass, Coronary artery bypass surgery, Heart team, Case report

## Abstract

**Background:**

Dislodgement of a coronary stent-balloon catheter during percutaneous coronary intervention (PCI) is rare but is a life-threatening complication.

**Case summary:**

A 57- year-old male presented with a non-ST elevation myocardial infarction (NSTEMI). Coronary angiography revealed total thrombotic occlusion of the Right coronary artery (RCA). Following the balloon dilatation of the RCA and while trying to retrieve the balloon catheter, the balloon was dislodged from the catheter shaft and entrapped in the coronary vessel. Under cardiopulmonary bypass, with antegrade cardioplegic arrest, the balloon was extracted through a coronary arteriotomy. Right coronary revascularization was done with reversed saphenous vein graft (SVG).

**Discussion:**

Given the variety of equipment that can be retained in the coronary artery and the multitude of mechanisms by which it may be entrapped, there are no straightforward techniques applicable to all situations. Specific guidelines or recommendations on properly managing these potentially life-threatening complications do not exist. However, the most crucial issue in the management of these cases is the hemodynamic status of the patient as well as the coronary flow in the vessel with entrapped device or stent. In our case, the RCA was retrogradely perfused from the left coronary artery, which provided time to transfer the patient to cardiovascular surgical backup.

## Introduction

On October 30, 1958, Mason Sones in Cleveland Clinic Foundation accidentally injected dye in a man’s coronary artery while performing aortography for a young patient with rheumatic heart disease. Placing milestones for cardiac catheterization. This was instrumental in developing coronary artery bypass surgery and interventional cardiology.

Percutaneous coronary intervention has developed tremendously over the last two decades and still contributes to saving millions of lives worldwide. It is nowadays a routine procedure in everyday cardiology practice. However, it can sometimes result in life-threatening complications. Balloon angioplasty can lead to different complications like coronary perforation and dissection of coronary arteries, which are potentially associated with significant morbidity and mortality. We report a case of entrapped coronary angioplasty balloon in the right coronary artery.

## Case presentation

A 57-year-old white male (1.8 m, 98 kg, BSA 2.18 m [2], BMI 30,2) presented to the Emergency Department (ED) in a secondary healthcare facility complaining of unstable angina pectoris. He was a chronic active smoker (40 peak years), and he reported previous treatment for high blood pressure and dyslipidemia. The past medical history was also pertinent for benign prostatic hyperplasia (BPH).

On presentation, blood pressure was 70/50 mmHg, heart rate 71 beats per minute, respiratory rate of 12 per minute, temperature 36,3 degrees Celsius using an ear thermometer, and oxygen saturation of 96% on ambient air.

A resting electrocardiogram revealed sinus rhythm, ST-segment depression on the precordial leads, and poor R-wave progression. High sensitivity Troponin upon admission was 700 pg/ml. A bedside transthoracic echocardiography revealed akinesis of the inferior wall of the left ventricle, left ventricular (LV) relaxation abnormality, and a mildly reduced left ventricular ejection fraction of 50%.

An early invasive strategy was decided, and the patient was administered aspirin and intravenous heparin. Prior to percutaneous coronary intervention, additional intravenous heparin was administrated to achieve an activated clotting time of more than 200 s.

An invasive coronary angiogram revealed thrombotic occlusion of the dominant right coronary artery (RCA). The left coronary system showed no significant diseases.

**Fig. 1:**
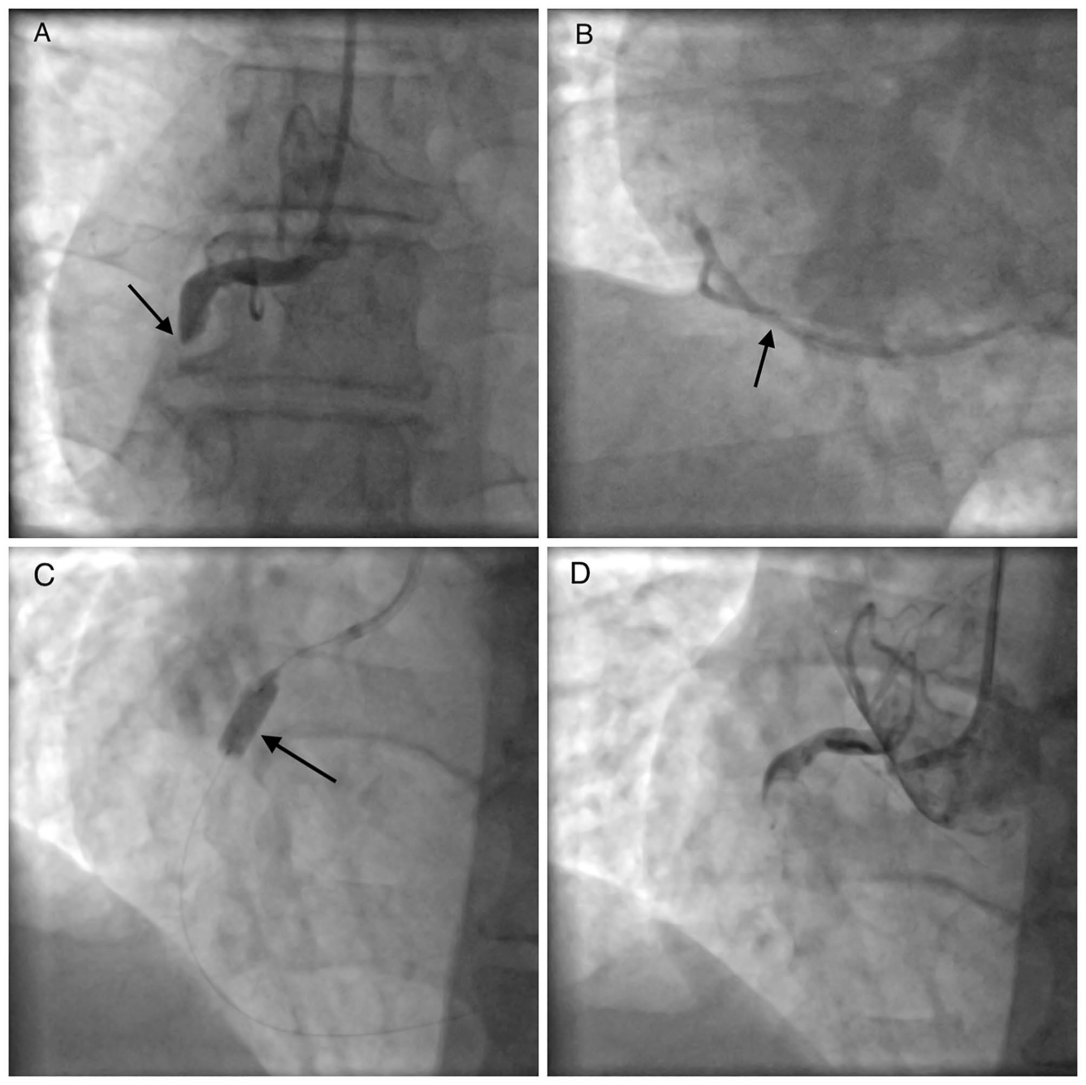
Angiographic images: **(A)** The baseline coronary angiography showed a thrombotic occlusion of the right coronary artery (RCA) (arrow); **(B)** Retrograde perfusion of the right coronary through the left coronary system (arrow); **(C)** Inflation of the balloon in the Right coronary artery (arrow); **(D)** Immediately after the procedure, dislodgment of the balloon from the shaft of the catheter and entrapment of it inside the right coronary artery

A PCI of the RCA was attempted, and a guidewire was inserted. An angioplasty balloon catheter was railroaded over it to place the stent in RCA after balloon dilatation. However, upon withdrawal of the balloon it was dislodged from the catheter shaft; it was then impacted in the RCA and couldn’t be pulled back. The patient was still hemodynamically stable as the right coronary artery was still retrogradely collateralized from the left coronary system **(**Fig. 1**)**. Angiography afterward showed no extravasation of the contrast material into the epicardial tissue. After an unsuccessful attempt to retrieve the balloon using an EN Snare system® (Merit Medical Systems, Inc.), the patient was swiftly transferred to our institution for urgent surgical intervention. Upon admission into our institution, high sensitivity troponin I was 22,654 pg/ml.

After induction of general anesthesia, routine perioperative transesophageal echocardiography (TOE) supported the diagnosis showing total inferior wall akinesia.

**Fig. 2:**
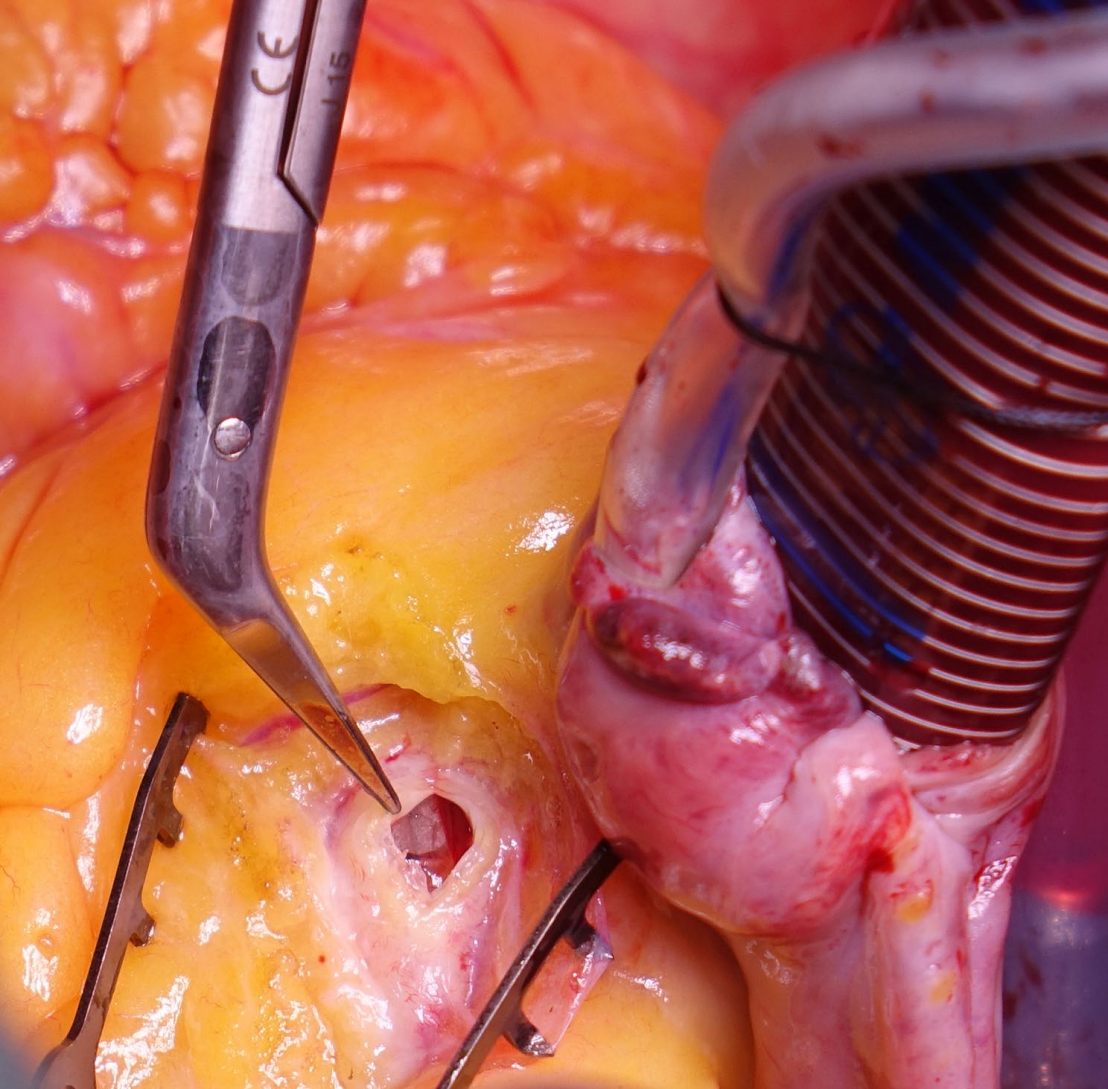
Intraoperative picture where the patient’s head is at 6 o’clock and legs at 12 o’clock, demonstrating the arteriotomy of the right coronary artery at the exact site of the entrapped balloon

**Fig. 3:**
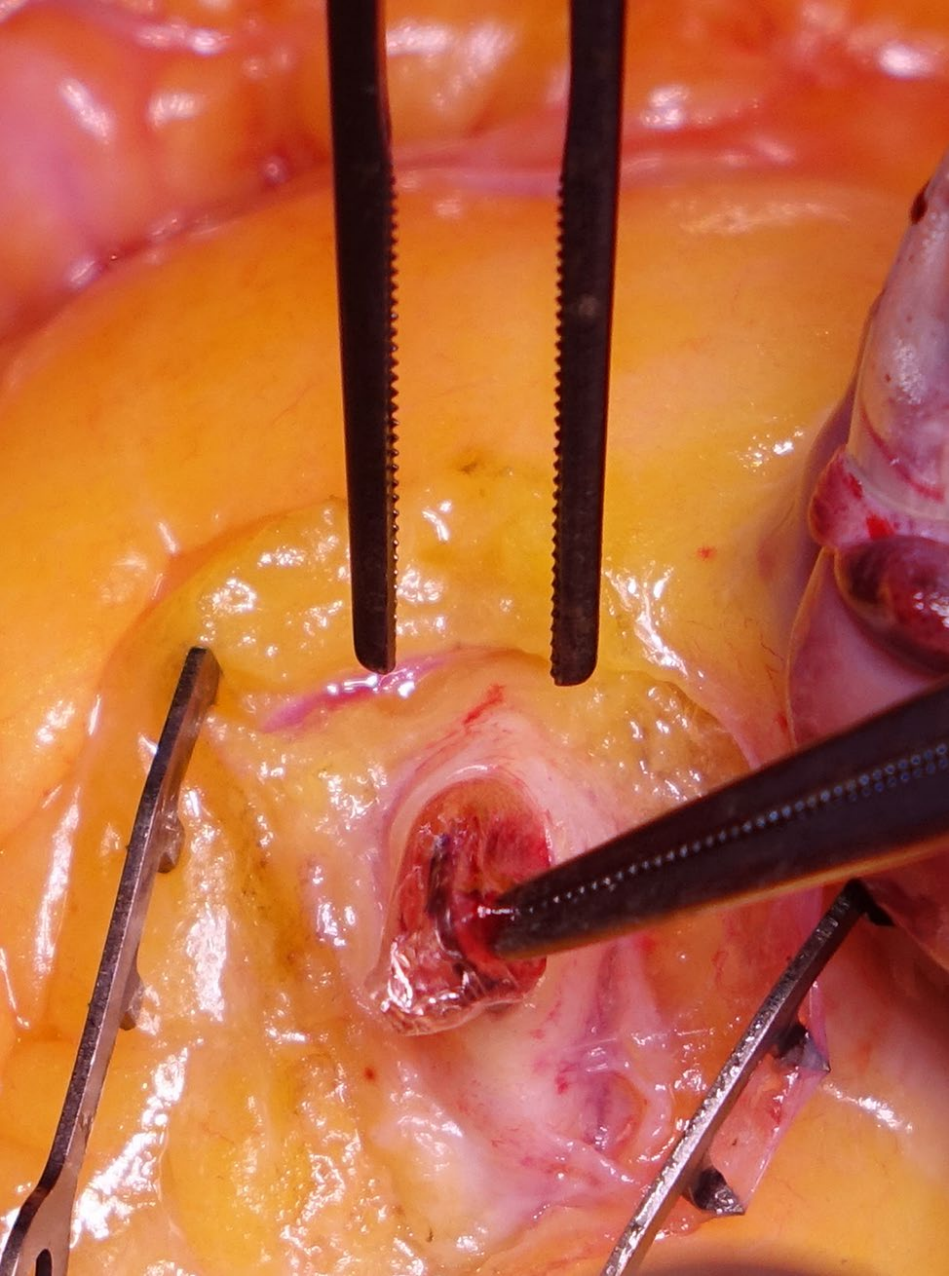
Intraoperative picture while pulling out the balloon

**Fig. 4:**
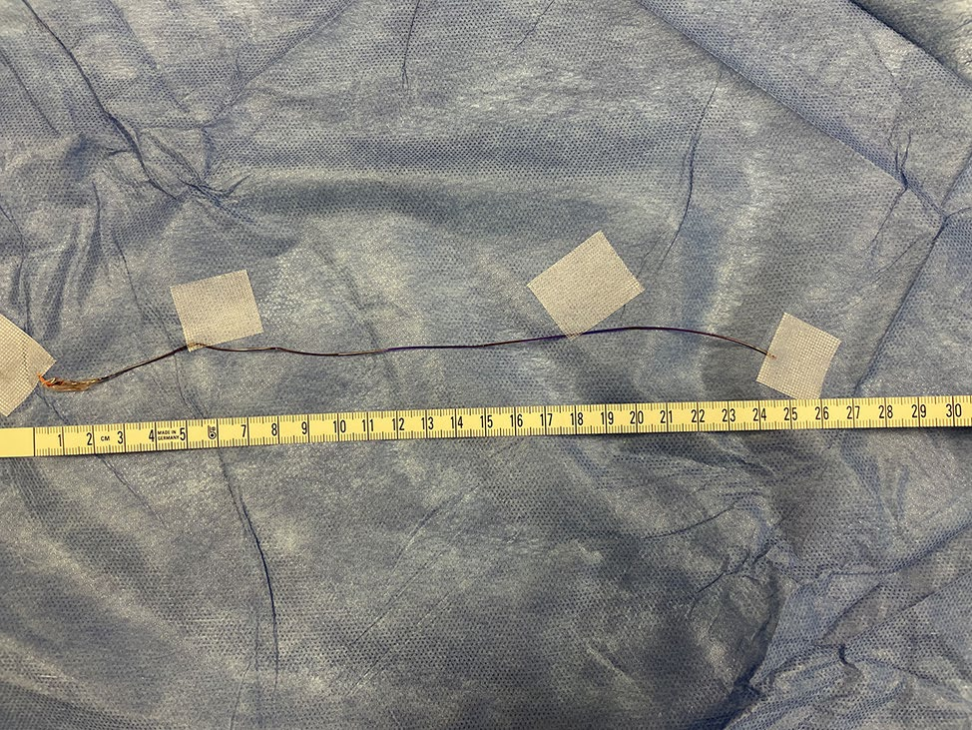
The approximate length of the extracted balloon in centimeters

**Fig. 5:**
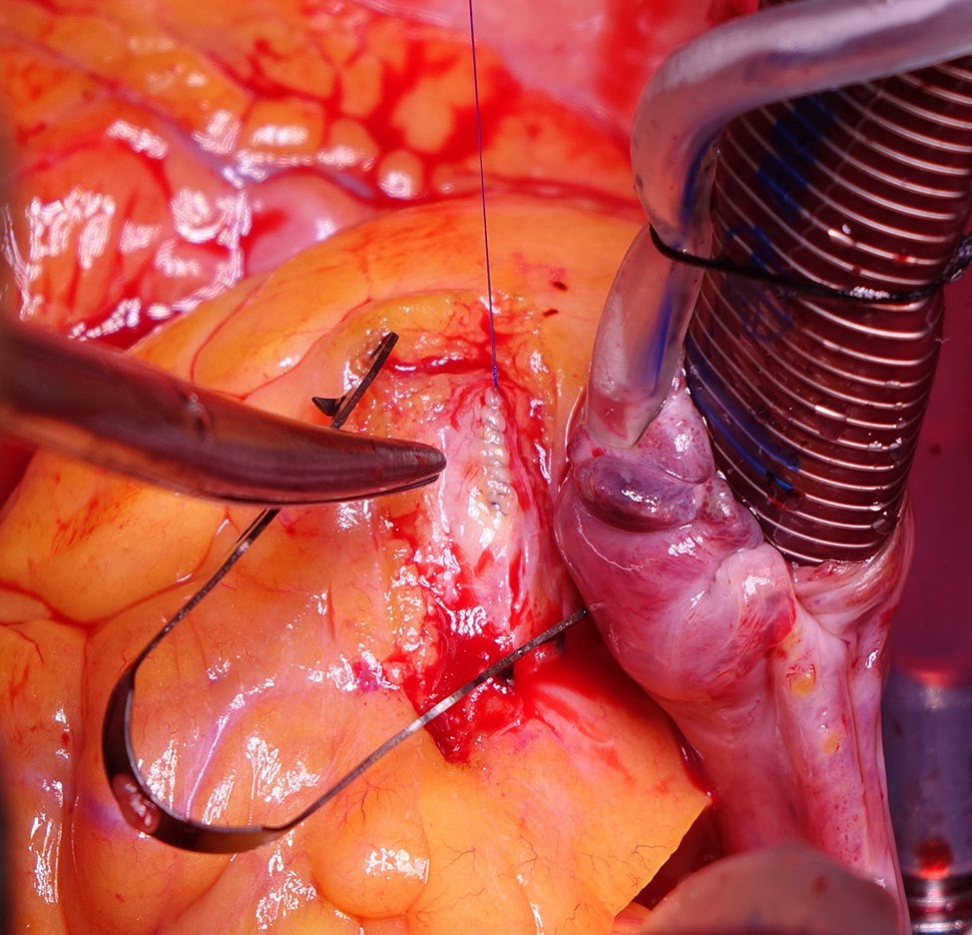
Intraoperative picture demonstrating Closing of the arteriotomy “pointed out with scissors.”

After median sternotomy, the patient was heparinized with checking activated clotting time. Aortic and right atrial cannulation was installed. A normothermic blood cardioplegia was administrated through the aortic root, which achieved a satisfactory diastolic arrest. A 2 mm probe was inserted distally in the native remaining RCA to assess the permeability. An isolated vein graft from the saphenous vein (SVG) was grafted to RCA, distal to the balloon entrapment site. A proximal right coronary arteriotomy was performed, and the balloon was pulled out **(**Figs. 2, 3 and 4**)**. Closing of the arteriotomy was utilized using a continuous Prolene 7.0 suture **(**Fig. 5**)**. The aortic root was vented. The heart was de-aired, and the aortic cross-clamp was removed. The proximal anastomosis was performed on a plaque-free area of the ascending aorta. The weaning off the cardiopulmonary bypass was performed as usual after the spontaneous return of heartbeats. The cardiopulmonary bypass was initiated for 51 min, and the aortic cross-clamp was applied for 28 min. Protaminized decannulation was done, and hemostasis was secured. The chest was then closed in layers.

The patient tolerated all the mentioned procedures well and was monitored in an intensive care unit for 48 h and then transferred to the surgical ward later for further postoperative care, which was uneventful. No neurologic event was observed postoperatively.

Follow-up transthoracic echocardiography showed normal heart dimensions and normal systolic function of both ventricles. The patient was discharged from the hospital on the 7th day postoperatively. He successfully recovered postoperatively with inpatient rehabilitation. Outpatient visit after four weeks was also uneventful.

## Discussion

Although the safety of percutaneous coronary interventions (PCI) has significantly improved over time, complications may still occur [1]. substantially impacting patient survival and healthcare costs. PCI procedural complexity and patient risk factors are increasing, and operators must be prepared to recognize and treat complications, including perforations, dissections, hemodynamic collapse, no-reflow, and entrapped equipment [2].

Procedural iatrogenic lesions occurring during percutaneous coronary intervention are of low incidence owing to this field’s augmented technical and procedural advancements over recent decades. On the other hand, these improvements have led interventional cardiologists to perform PCI in patients with higher-risk anatomic features, such as chronic total occlusions, bifurcations, and left main lesions. The overall comorbid complexity of PCI patients has also increased over recent years, and percutaneous procedures are nowadays performed in older patients with more frequent comorbidities and heavily calcified coronary arteries [3]–[4]. Percutaneous cardiac interventions are also exponentially expanding into the valvular and electrophysiological fields. Consequently, iatrogenic lesions to cardiac structures will continue to be possible [3].

Surgical outcomes in these groups of patients have a high incidence of morbidity and mortality due to severe ongoing myocardial ischemia and hemodynamic impairment, with compromised anatomic integrity, thus often requiring mechanical ventilation and high-dose inotropic support or even cardiopulmonary resuscitation. It must also be considered that because of their emergent nature, these surgical procedures lack presurgical evaluation and adequate preoperative management. In addition, most of these patients receive a loading dose of antiaggregants or have not discontinued anticoagulation, and many may be actively bleeding. This, in turn, results in a high risk of severe bleeding and subsequent multiple blood product transfusions, which may contribute to the transmission of a variety of blood-borne infections (BBIs), allergic reactions, and multiorgan failure [5].

Various management modalities have been introduced as bailout strategies in case of device entrapment in the coronaries, such as snaring, pulling, and telescoping techniques. In a systemic literature review [6], it was alleged that efforts to avoid surgical intervention should be maximized as the outcome might be inferior to other strategies due to the lack of expertise to deal with such a rare incident. The surgery in this patient was completed successfully, and the patient could be discharged with retained myocardial functions. More studies are needed in the future to evaluate the long-term outcome.

The timing of emergency surgery constitutes another critical aspect. As most patients who require surgery after iatrogenic lesions present with poor clinical status, the time interval between the occurrence of the injury and the surgical treatment must be minimized, as mortality was reported to be significantly higher in patients with more extended time intervals between iatrogenic PCI event and skin incision [7], so in case of nonemergent PCI, it would be wise to transfer patients with a complex coronary disease to a hospital with on-site cardiac surgery backup.

Predicting a potential complication is of utmost importance in minimizing iatrogenic injuries after a percutaneous procedure. This can be realized by identifying risk factors and high-risk anatomies through accurate, thorough, and multidisciplinary preprocedural evaluation of patients [8]; This emphasizes the role of the Heart Team in properly selecting and planning the best treatment strategy when a high-risk procedure is identified. For example, a surgical backup or a hybrid approach can be set up, or mechanical circulatory support systems, such as venoarterial extracorporeal membrane oxygenation or mechanical left ventricle assistance, can maintain the adequate hemodynamic status [9].

## Conclusion

This case highlights the importance of the Heart-Team concept and its contribution to raising the standards of care in patients with cardiovascular diseases.

**Learning points**:

- Modern cardiovascular care revolves around the concept of a heart team.
- Complex coronary disease patients undergoing non-emergent PCI should be transferred to a facility with on-site cardiac surgery backup.
- Failure to implement a Heart Team is becoming increasingly infeasible, as the Heart Team is mandated in certain clinical situations by societal guidelines, and reimbursement for associated procedures has been tied to its implementation. To optimize patient-centered care, healthcare systems will need to evolve to incorporate these teams.

## Data Availability

The raw data supporting the conclusions of this article will be made available by the authors without undue reservation to any qualified researcher.
